# Fixation probability of a beneficial mutation conferring decreased generation time in changing environments

**DOI:** 10.1186/s12918-018-0575-9

**Published:** 2018-04-24

**Authors:** Fangshu Cui, Bo Yuan

**Affiliations:** Department of Computer Science and Engineering, Shanghai Jiao Tong University, Shanghai, 200240 China

**Keywords:** Fixation probability of beneficial mutations, Selective advantage, Branching process, adaptation

## Abstract

**Background:**

One central building block of population genetics is the fixation probability. It is a probabilistic understanding of the eventual fate of new mutations. Moreover, the fixation probability of new beneficial mutations plays an important effect on the adaptation of populations to environmental challenges. Great progress has been made in the study of the beneficial mutations that increases offspring number. However, the fixation probability of beneficial mutations with a shorter generation time under various genetic and ecological conditions has not been explored.

**Results:**

Here we extend the classical result of the fixation probability of beneficial mutations obtained by Haldane, and estimate the fixation probability of a beneficial mutation with a reduced generation time in a changing environment. Assuming that the selective advantage is very small, we concentrate all the changing factors of environment on a single quantity: effective selective advantage. Using a time-dependent branching process, we get the analytic approximation for the fixation probability of beneficial mutations that decrease the generation time. Then, we apply this approximation to four interesting biological cases.

**Conclusions:**

In these instances, we show the comparison of the approximation with the accurate values. We find that they are consistent, demonstrating the effectiveness of our result for the fixation probability of beneficial mutations conferring a reduced replication time.

## Background

One cornerstone of population genetics is the fixation probability, i.e. the probability that mutations survive loss. This is a probabilistic comprehension of the eventual fate of the beneficial, neutral, or deleterious mutations. Furthermore, the fixation probability of new beneficial mutations has a significant impact on the rate of adaptation of populations [1, 2]. When a new beneficial mutation goes into a population, to finish an adaptive step, it has to escape random loss due to genetic drift, rise to enough copies, and eventually get fixed. Actually, the frequency of a beneficial mutation fluctuates over time. When this frequency is low, the genetic drift is likely to lead to the disappearance of the beneficial mutation, which needs a stochastic process. Once this frequency is large enough, a deterministic model can be used to well approximate the further increment of this frequency. Usually, the calculation for the frequency of a beneficial mutation is equivalent to evaluating the probability that the beneficial mutation survives an earliest stage of strong genetic drift.

Since 1920s, interest in the computation of fixation probabilities has been maintained for nearly one century and considerable progress has been made in this problem. Generally, there are three methods to estimate the fixation probabilities: Markov chain, branching process and diffusion approximation. When the individuals and genotypes in a population can be enumerated, the Markov chain method can obtain the fixation probability precisely. Therefore, this method is characteristically practicable only when the population size is very small [3, 4]. Once the population size becomes large, the discrete branching processes are in wide use [5–13]. Since the branching process method presumes that the population is large enough that the destiny of each mutation is independent of all others, it gives an approximation to the real fixation probability. When the selection is weak in a large population, the diffusion approximation approaches are usually used [9, 14–16]. Furthermore, many literatures have tried to integrate and reconcile the discrete and continuous methods [8, 9, 17, 18].

Classically, if a native wild-type individual has on average one offspring per generation, a beneficial mutant has on average 1 + *s* offspring per generation, where the parameter *s* (*s* > 0) is the selective advantage. This mechanism of the selective advantage is defined as fecundity, which is fundamental in a large amount of literature in population genetics [19–22]. Assuming a Poisson offspring distribution and a small, constant selection coefficient in a population of constant size, Haldane [20] gives the well-known result that the fixation probability is approximately 2*s*, for a mutation that increases fecundity.

Nevertheless, the mutants in many organisms may produce the same number of offspring as the wild-type in a shorter generation time: so-called “generation time” mutants. For example, in the bacteria population, a mutant that has the antibiotic resistance completes the cell cycle and produces two offspring faster than the drug-sensitive individuals. In this case, a reduced replication time is obviously a more suitable mechanism for the selective advantage. Wahl and DeHaan [12] have firstly demonstrated that the classic approximation 2*s* for the fixation probability of a beneficial mutation does not hold for this mutation conferring a decreased development time. Using a model with a Poisson-distributed offspring with mean 2 and a weak, constant selective advantage, they have approximated the fixation probability of this “generation time” mutant as *s*/ln(2) for a population of constant size. Therefore, if all mutations are assumed to increase the offspring number, it leads to an overestimate of the order 2 ln(2) for the fixation probability of the mutation that reduces the replication time.

The study of fixation probability under diverse genetic factors and ecological scenarios has been explored [23]. A series of articles have estimated the survival probability of beneficial mutations when the population size changes [3, 4, 8, 9, 18, 24]. Ewens [24] derived the fixation probability of a beneficial mutation in two cases of changing population sizes: a cyclic sequence of population sizes and the population size that first increases and then remains constant. Otto and Whitlock [8] studied the survival probability of beneficial mutants under several demographic models of population size change, including a single change, exponential growth or decline, logistic growth or decline, and fluctuating size. Wahl and Gerrish [9] examined the influence of population bottlenecks on the fixation probability. Lambert [18] and Parsons and Quince [3, 4] developed the fixation probability of beneficial mutations when the population size changed dynamically. Since Pollak [25] first studied the fixation probability in a subdivided population, great process has been made in this probability in spatially heterogeneous populations [16, 26–28]. The influence of linked loci on the fixation probability of an advantageous allele has been widely investigated [29–32]. Studies on time-dependent selection mainly focus on random fluctuations of selection coefficients [33–36]. Recently, Waxman [37] and Uecker and Hermisson [38] addressed the question of the establishment of new beneficial mutants when the change of selection coefficients and population sizes follows an explicit trend. Peischl and Kirkpatrick [39] derived analytical approximations for the fixation probability of favorable mutations in arbitrarily changing environment that used a novel approach assuming small environmental fluctuations. In these studies, the beneficial mutations are assumed to increase the average number of offspring. However, when a mutant confers an advantage in generation time, the effects of various genetic and ecological conditions on the fixation probability have not received enough attention.

In this article, we use a time-dependent branching process to study the fixation probability of “generation time” mutants in changing environments. Assuming weak selection, we centralize all the environmental changes into a single parameter: effective selective advantage, and deduce an analytical approximation for this fixation probability. We apply our result to four absorbing biological cases, including the monotone increase and the periodic change of the selective advantage in a population of constant size, the changing population size, and the stochastic fluctuations in selection. In these conditions, our approximation compares well with the numerical calculation, which demonstrates the effectiveness of our result.

## Methods

### Branching process

In probability theory, the branching process is a mathematical object known as stochastic process. It is used to model reproduction, that is, to model a population in which each individual produces stochastic number of offspring in the next generation. It can also be used to model some other similar dynamics, for example, the dispersion of surnames in genealogy, the spread of neutrons in a nuclear reactor and so on.

**Fig. 1:**
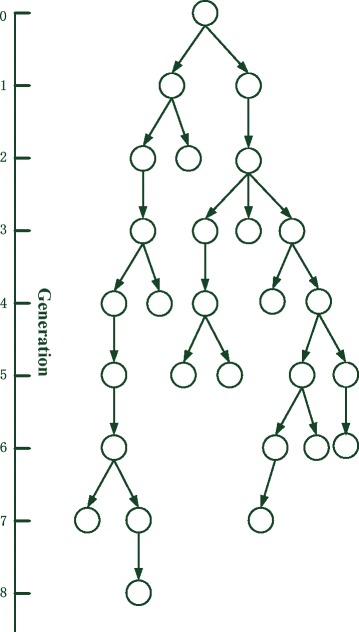
The lineage of an individual with a branching process model. Originally, at generation *t* = 0 there is a single individual. At each generation, each individual produces *k* offspring with the assigned probability *f*_*k*_. In this condition, the lineage becomes extinct after *t* = 8 generations

In a discrete-time branching process (Fig. 1), each individual in a population produces *k* (*k* = 0, 1, 2, ...) offspring with the assigned probability *f*_*k*_ in the next generation. Then, these offspring have the same reproductive capacity with the ancestor, that is, each of them produce *k* offspring with the probability *f*_*k*_, and so on. The crucial hypothesis of the branching process model is that the offspring distribution of each individual is independent and identical. However, only when the mutant lineage is a small part of the population, this hypothesis is true. If the population size is constant and the mutant lineage becomes a large part of the population, the density of each individual must rely on the others.

### Fixation probability of beneficial mutations in a constant environment

Assuming a wild-type individual in a population has *r* offspring on average per generation, this individual will have *r*^*t*^ offspring and a “fecundity” mutant having selective advantage *s* will have (*r*(1 + *s*))^*t*^ offspring in *t* generations. In the “growth” stage of a population, a lot of offspring are reproduced. Due to the assumption of constant population size, only part of offspring can survive in the next generation, which is called the “sampling” stage. We use *y*(*x*) to be the probability generating function that represent the number of offspring reproduced by a “fecundity” mutant lineage in one generation, that is, the probability generating function in the “growth” stage. The *y*(*x*) is described as follows: if the probability that an individual produces *i* offspring is *p*_*i*_, *y*(*x*) = *p*_0_ + *p*_1_*x* + *p*_2_*x*^2^ + ... [40]. Assuming that the offspring obey the Poisson distribution and the average number of offspring of a wild-type individual is *r* = 2, a mutant with a fecundity advantage *s* has 2(1 + *s*) offspring on average per generation. Hence,

1 $$ {\displaystyle \begin{array}{l}y(x)={p}_0+{p}_1x+{p}_2{x}^2+\cdots \\ {}\kern1.00em =\sum \limits_{k=0}^{\infty}\frac{\exp \left[-2\left(1+s\right)\right]{\left[2\left(1+s\right)\right]}^k}{k!}{x}^k\\ {}\kern1.00em =\exp \left[2\left(1+s\right)\left(x-1\right)\right]\end{array}} $$

We use *N* to denote the population size, which involves both the wild type and the mutant. Because the population size is constant, only *N* of the newly produced offspring will survive in every generation. Owing to the supposition that the average number of offspring of each wild-type individual is *r*, the probability that each offspring survives is 1/*r*. Conversely, the probability that each offspring dies is 1 − 1/*r*. Therefore, the probability generating function in the “sampling” stage is *z*(*x*) = 1 − 1/*r* + (1/*r*)*x*. According to the property of the probability generating function, the overall probability generating function of “growth” stage followed by “sampling” stage is *ϕ*(*x*) = *y*(*z*(*x*)) = *y* ∘ *z*(*x*), which is the total probability generating function for the number of offspring in a “fecundity” mutant lineage after one generation. Consequently, the whole probability generating function for the “fecundity” mutant lineage after *n* generation is

2 $$ {\phi}_n(x)=\phi \circ \phi \circ \phi \circ \cdots \left(n\kern0.5em times\right)\cdots \circ \phi (x) $$

By calculating the value of the probability generating function at *x* = 0, we can eliminate the higher terms and obtain the probability that the mutant lineage eventually disappear. Hence, the extinction probability of a “fecundity” mutation is $q=\underset{n\to \infty }{\lim }{\phi}_n(0)$. And, the fixation probability of the “fecundity” mutation is [20].

3 $$ p=1-\underset{n\to \infty }{\lim }{\phi}_n(0) $$

The deduction of the fixation probability above can be expanded to the case that a population has population bottlenecks. We suppose that the bottlenecks happen every *τ* generations, where *τ* is constant. The “fecundity” mutant lineage will experience *τ* sequential “growth” stages and one “sampling” stage, so the total probability generating function is

4 $$ y(x)=y\circ y\circ y\circ \cdots \left(\tau \kern0.5em times\right)\cdots \circ y\circ z(x) $$

Here, the *ϕ*(*x*) can be simply denoted as *ϕ*(*x*) = *y*_*τ*_ ∘ *z*(*x*). Particularly, the situation that the bottlenecks occur every *τ* = 1 generations is equal to a constant population size.

We will give the calculation method of the fixation probability of a “generation time” mutation below [12]. Let *t*_*g*_ (*t*_*g*_ < 1) be the generation time of the “generation time” mutant lineage. In *t* wild-type generations, a “generation time” mutant will produce ${r}^{t/{t}_g}$ offspring on average. Assuming that the whole growth rate of both the “fecundity” mutant lineage and the “generation time” mutant lineage is the same, we have ${r}^{t/{t}_g}={\left(r\left(1+s\right)\right)}^t$ and obtain

5 $$ {t}_g=\frac{1}{1+{\log}_r\left(1+s\right)} $$

Let $\tilde{s}={\log}_r\left(1+s\right)$, then ${t}_g=1/\left(1+\tilde{s}\right)$. When *s* is very small, we have $\tilde{s}\approx s/\ln (r)$.

For the “generation time” mutation, the mutant lineage will finally undergo *τ* + 1 generations between two population bottlenecks. If a mutation of this type firstly appears at the start of a “growth” stage, the situation above will firstly happen before *n*_1_ population bottlenecks. Therefore, we have

6 $$ \left({n}_1\tau +1\right)\left(\frac{1}{1+\tilde{s}}\right)\le {n}_1\tau $$

and obtain

7 $$ {n}_1\ge 1/\left(\tilde{s}\tau \right) $$

Extending the Eq. (6), the situation that the “generation time” mutant lineage undergo an extra generation occurs before *n*_*i*_ consecutive population bottlenecks, we find $\left({n}_i\tau +i\right)\left[1/\left(1+\tilde{s}\right)\right]\le {n}_i\tau$ and have ${n}_i\ge i/\left(\tilde{s}\tau \right)$.

When the “generation time” mutant lineage experiences an extra generation, the probability generating function of “growth” stage followed by “sampling” stage is *ϕ*^+^(*x*) = *y*_*τ* + 1_ ∘ *z*(*x*). For all the other “growth” and “sampling” stages, the probability generating function is *ϕ*(*x*) = *y*_*τ*_ ∘ *z*(*x*). For instance, if *τ* = 5, $\tilde{s}=1/20$, we obtain *n*_1_ ≥ 4. Thus, the probability generating function of this mutant lineage after eight population bottlenecks is

8 $$ {\phi}_8(x)=\phi \circ \phi \circ \phi \circ {\phi}^{+}\circ \phi \circ \phi \circ \phi \circ {\phi}^{+} $$

Accordingly, the fixation probability of the “generation time” mutation can be computed by the Eq. (3).

### Fixation probability of beneficial mutations in a variable environment

We explore the fixation probability of beneficial mutations when the selective advantage *s*_*k*_ (*s*_*k*_ > 0, and *s*_*k*_ ≪ 1, *k* = 1, 2, 3,...) changes in time in a changing environment. Extending the classical result for the fixation probability of beneficial mutations in a constant environment, Peischl and Kirkpatrick [39] have provided this probability in a time-dependent branching process:

9 $$ 1-p=\underset{n\to \infty }{\lim }{\phi}_0\left({\phi}_1\left(\cdots {\phi}_n(0)\right)\right) $$

Combined with the probability generating function of the “generation time” mutant given above, the fixation probability of this mutant lineage in changing environments can be evaluated by the Eq. (9). Due to the computational complexity of the nested structure in Eq. (9), we need a simple analytic approximation to describe this probability. Peischl and Kirkpatrick [39] have given an approximation of the fixation probability of a “fecundity” mutation in a variable environment:

10 $$ p\approx 2{s}_e $$

where the offspring obey the Poisson distribution and *s*_*e*_ is the effective selective advantage.

Here we aim to develop an analytic approximation for the fixation probability of “generation time” mutations in changing environments using a time-dependent branching process. Firstly, we innovate a reference environment. In this reference environment, assuming a Poisson-distributed offspring and a small, constant selection coefficient $\overline{s}$ ($\overline{s}>0$) in a population of constant size, we get the fixation probability of the “generation time” mutant as $\overline{p}=\overline{s}/\ln (2)$ [12]. Let $\overline{\phi}$ be the probability generating function of the “generation time” mutant in this reference environment, we define the probability generating function of this mutant lineage at generation *k* in a variable environment as:

11 $$ {\phi}_k(x)=\overline{\phi}(x)+{\varepsilon}_k(x) $$

where *ε*_*k*_, *k* = 1, 2, 3, ... is the disturbance function in generation *k*. *ε*_*k*_ is a smooth and bounded function that maps [0,1] to [− 1,1], and *ε*_*k*_(1) = 0, *k* = 1, 2, 3, .... Assuming that the offspring obey the Poisson distribution and its instantaneous variation is very small in the changing environment, we have max_*x*, *k*_[*ε*_*k*_(*x*)] ≪ 1, ${\max}_{x,k}\left[{\varepsilon}_k^{\prime }(x)\right]\ll 1$. We substitute the Eq. (11) into the Eq. (9) and expand in a Taylor series

12 $$ p=\overline{p}-\sum \limits_{k=0}^{\infty }{\varepsilon}_k\left(1-\overline{p}\right){\sigma}^k+O\left({\varepsilon}^2\right) $$

Here, $\sigma ={\overline{\phi}}^{\prime}\left(1-\overline{p}\right)$, and *O*(*ε*^2^) denotes the order of max_*x*, *k*_[*ε*_*k*_^2^(*x*)] and ${\max}_{x,k,l}\left[{\varepsilon}_k(x){\varepsilon}_l^{\prime }(x)\right]$. The average number of offspring in the reference environment is more than 1, so we have 0 < *σ* < 1. Because *ε*_*k*_ is bounded, the ${\sum}_{k=0}^{\infty }{\varepsilon}_k\left(1-\overline{p}\right){\sigma}^k$ in the Eq. (12) is convergent for every series of environments.

Because the selective advantage *s*_*k*_ of the “generation time” mutation in variable environments is very small, i.e. *s*_*k*_ ≪ 1, *k* = 1, 2, 3, ..., and $\overline{s}\ll 1$, we have

13 $$ \sigma \approx {\overline{\phi}}^{\prime}\left(1-\frac{\overline{s}}{\ln 2}\right)\approx {\overline{\phi}}^{\prime }(1)-{\overline{\phi}}^{{\prime\prime} }(1)\frac{\overline{s}}{\ln 2}\approx 1-\overline{s} $$

Neglecting the second order terms and high order terms of (*x* − 1), we obtain an approximation of the disturbance function *ε*_*k*_

14 $$ {\varepsilon}_k(x)\approx {\varepsilon}_k(1)+{\varepsilon}_k^{\prime }(1)\left(x-1\right)=\left({s}_k-\overline{s}\right)\left(x-1\right) $$

Substituting the Eq. (14) into the Eq. (12), one finds

15 $$ p\approx \overline{p}+\overline{p}\sum \limits_{k=0}^{\infty}\left({s}_k-\overline{s}\right){\sigma}^k=\overline{p}\sum \limits_{k=0}^{\infty }{s}_k{\left(1-\overline{s}\right)}^k $$

We define

16 $$ {\omega}_k=\overline{s}{\left(1-\overline{s}\right)}^k $$

Since ${\sum}_{k=0}^{\infty }{\omega}_k=1$, {*ω*_*k*_}can be understood as a probability distribution. And, the value of *ω*_*k*_ diminished over time. Here we define the effective selective advantage *s*_*e*_ as:

17 $$ {s}_e=\sum \limits_{k=0}^{\infty }{\omega}_k{s}_k $$

So the *s*_*e*_ can be regarded as a weighted average. Consequently, in a variable environment where the selective advantage changes over time, an analytical approximation for the fixation probability of “generation time” mutations is given by

18 $$ p\approx \frac{s_e}{\ln 2} $$

In the derivation above, it is important how to select an appropriate reference environment. Generally, we use the arithmetic average of the selective advantage to define the reference environment

19 $$ \overline{s}=\underset{n\to \infty }{\lim}\left(\frac{1}{n}\sum \limits_{k=0}^n{s}_k\right) $$

## Results

We apply our analytic approximation to four interesting biological instances of changing environments. In these cases, we compare our approximation for the fixation probability of “generation time” mutations (Eq. (18)) to the exact value acquired by numerical iteration of Eq. (9).

### Monotonously increasing selection

We assume that the selective advantage of a “generation time” mutation monotonously increases from *s*_0_ to *s*_∞_ in a population of constant size:

20 $$ {s}_k={s}_0{e}^{-\frac{1}{2}k}+{s}_{\infty}\left(1-{e}^{-\frac{1}{2}k}\right) $$

The selective advantage of reference environment obtained by the Eq. (19) is

21 $$ \overline{s}=\underset{n\to \infty }{\lim}\left(\frac{1}{n}\sum \limits_{k=0}^n\left[{s}_0{e}^{-\frac{1}{2}k}+{s}_{\infty}\left(1-{e}^{-\frac{1}{2}k}\right)\right]\right)={s}_{\infty } $$

Assuming *s*_∞_ ≪ 1, we have ${e}^{-{s}_{\infty }}\approx 1-{s}_{\infty }$. The effective selective advantage acquired by the Eq. (17) is

22 $$ {s}_e\approx {s}_{\infty}\left[1+\frac{s_0-{s}_{\infty }}{1-{e}^{-\frac{1}{2}}\left(1-{s}_{\infty}\right)}\right] $$

In this case, the fixation probability of “generation time” mutations can be approximated as

23 $$ p\approx \frac{s_{\infty }}{\ln 2}\left[1+\frac{s_0-{s}_{\infty }}{1-{e}^{-\frac{1}{2}}\left(1-{s}_{\infty}\right)}\right] $$

**Fig. 2:**
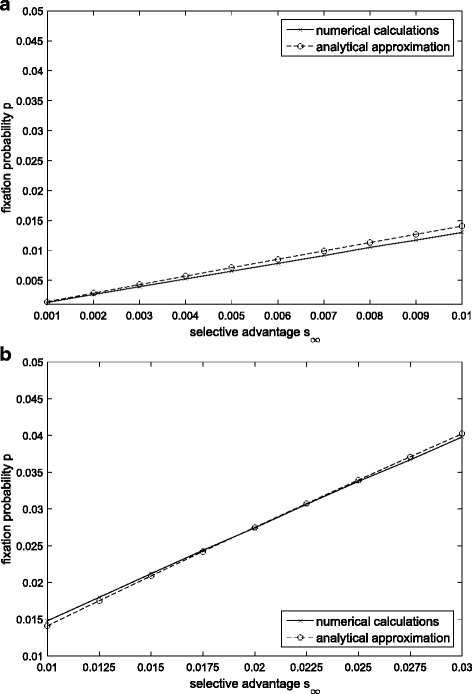
Comparison of the approximation with exact results in the case of monotonously increasing selection. The initial selective advantage is *s*_0_ = 0 in the figure (**a**), and *s*_0_ = 0.001 in the figure (**b**)

In the Fig. 2, we compare our approximation with the numerical computations in this instance, and find that they are consistent. Accordingly, the analytic result we deduced is a good approximation for the fixation probability of “generation time” mutations.

### Cyclically changing selection

In the natural environment, a population usually goes through periodic environmental changes, such as seasonal variations in the temperature and humidness. Assume that the selective advantage of a “generation time” mutation experiences periodic changes as follows

24 $$ {s}_k={s}_{mean}+\varDelta s\cos \left( k\rho +\theta \right) $$

where *s*_*mean*_ is the mean of selective advantage, *Δs* is the amplitude of fluctuations, *ρ* is a parameter that controls the length of a circle of fluctuations, and *θ* is used to decide the initial selective advantage.

The selective advantage of reference environment is

25 $$ \overline{s}=\underset{n\to \infty }{\lim}\left(\frac{1}{n}\sum \limits_{k=0}^n\left[{s}_{mean}+\varDelta s\cos \left( k\rho +\theta \right)\right]\right)={s}_{mean} $$

The effective selective advantage is

26 $$ {s}_e\approx {s}_{mean}\left[1+\varDelta s\frac{\left(1-{s}_{mean}\right)\cos \left(\rho -\theta \right)-\cos \theta }{2\left(1-{s}_{mean}\right)\cos \rho -{\left(1-{s}_{mean}\right)}^2-1}\right] $$

In this instance, the analytic approximation for the fixation probability of “generation time” mutations is

27 $$ p\approx \frac{s_{mean}}{\ln 2}\left[1+\varDelta s\frac{\left(1-{s}_{mean}\right)\cos \left(\rho -\theta \right)-\cos \theta }{2\left(1-{s}_{mean}\right)\cos \rho -{\left(1-{s}_{mean}\right)}^2-1}\right] $$

**Fig. 3:**
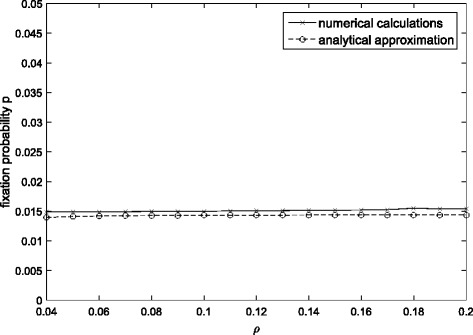
Comparison between the approximation and exact values when the selective advantage periodically changes. The parameter values are *S*_*mean*_ =0.01, Δs =0.005, and *θ = π*

Figure 3 shows the comparison of the approximation with the accurate value in the case of cyclically changing selection. It can be seen that our approximation gives an exact prediction for the fixation probability of “generation time” mutations.

### Varying population size

We assume that the selective advantage of the “generation time” mutants *s* is constant in a population in which the population size changes over time. The average number of offspring of each wild-type individual in generation *n* is *N*_*n* + 1_/*N*_*n*_, and that of each mutant is

28 $$ {M}_n=\frac{N_{n+1}}{N_n}\left(1+s\right) $$

where *N*_*n*_ is the population size of the wild-type individuals in generation *n*. We use the solution of the Beverton-Holt equation as the demographic dynamics of the wild-type individuals:

29 $$ {N}_n=C\frac{N_0}{N_0+\left(C-{N}_0\right){e}^{- gn}} $$

Here, *N*_0_ denotes the initial population size, *g* denotes the growth rate of the population, and *C* denotes the carrying capacity.

**Fig. 4:**
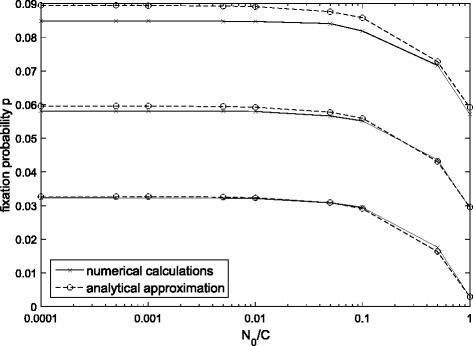
Comparison of the approximation with accurate results when the population size changes. The parameter values are *s* = 0.02, 0.01, and 0.001 (from top to bottom) and *g* = 0.01

In the Fig. 4, a comparison between the approximation and accurate results is shown. In this instance, our approximation matches well with the exact values, which shows our result is effective.

### Stochastic fluctuations in selection

Suppose the selective advantage of a “generation time” mutation experiences random fluctuations as

30 $$ {s}_k=\left(1-\lambda \right){s}_{mean}+\lambda {s}_{k-1}+{\xi}_k $$

where *s*_*mean*_ is the mean of selective advantage, *λ* ∈ [0, 1] is the correlation coefficient between *s*_*k* − 1_ and *s*_*k*_, and *ξ*_*k*_ is a white noise that has mean 0 and variance *σ*^2^.

If this process has a condition that the initial selective advantage is *s*_0_, it becomes time dependent and the expected selective advantage in generation *k* is

31 $$ E\left[{s}_k|{s}_0\right]=\left(1-{\lambda}^k\right){s}_{mean}+{\lambda}^k{s}_0 $$

Thus, the expected fixation probability of “generation time” mutations is

32 $$ E\left[p|{s}_0\right]=\frac{s_{mean}}{\ln 2}\left[1+\frac{s_0-{s}_{mean}}{1-\lambda \left(1-{s}_{mean}\right)}\right] $$

**Fig. 5:**
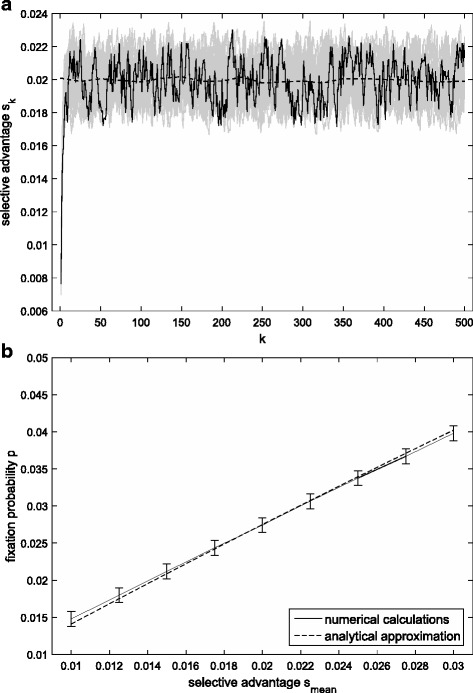
**a** Examples of Eq. (30). The solid curve shows one example, and shaded curves show 20 other examples. The dashed line shows the mean selective advantage. The parameter values are *s*_0_ = 0.001, *s*_*mean*_ = 0.02, *λ* = 0.6, and *σ* = 0.001. **b** Comparison between the approximation and exact results when the selective advantage experiences stochastic fluctuations. The whiskers show 99% confidence intervals. The other parameter values are as in the top figure

Figure 5 shows the comparison of the approximation with the accurate value in the case of random fluctuations in selection. It can be seen that they are coincident, which proves the effectiveness of our approximation.

## Discussion

In the nature, if an organism carries a beneficial mutation, it will propagate more quickly than the wild-type individuals. In a large number of literature, this means that the beneficial mutant can reproduce more surviving offspring in each generation, i.e. “fecundity” mutant. Nevertheless, a great deal of organisms may produce the same number of offspring as the wild-type in a shorter generation time, i.e. “generation time” mutant. In many cases, the mechanism of the beneficial mutations is very important. Therefore, we studied the fixation probability of “generation time” mutations in variable environments that has not been explored.

The adaptation of populations to environmental challenges relies on the fixation probability of new beneficial mutations. There are a variety of origins of environmental change, and variations can happen in all time scales, from transient variations to transitions in the geological time scales. However, a majority of researches of adaptation depend on the detachment of time scales in the evolution and ecology. This is maybe a serious simplification in many situations. Thus, we explored the establishment probability of beneficial mutations in changing environments.

In a population, individual alleles can undergo changing selection pressures even though the outside environment is constant. This is because multiple selected alleles separate and interfere owing to the linkage or the epistasis [41]. There are many examples, such as the evolution of the compensatory mutation, clonal interference, adaptive genes that flow across a genetic barrier, and so on.

In population genetics, the deduction of the fixation probability from branching processes is a common method. Using branching processes, it is easy to obtain simple analytic results, which are precise when the population size is large enough that *Ns* ≫ 1. The shortcoming of this method is that it is only applicable to beneficial and originally rare mutations. So far, branching processes have been also applied to some other natural phenomenon, including the spread of communicable diseases, the increment of tumor cells and so on [42, 43].

To show how our analytic approximation can be applied to special instances of variable environments, we used it to a few biological cases. They are the monotonously increasing selection, periodically changing selection, changing population size and random fluctuations of selection. In these examples, in order to guarantee the positive result of Eq. (9), we focused on the scenarios that the selective advantage *s*_*k*_ > 0. Generally, our approximations match well with the accurate values (see Figs. 2-5). Nevertheless, our result underestimates the fixation probability of “generation time” mutations if ${s}_k\ll \overline{s}$ in initial generations (see Fig. 5).

The theoretical framework can be tested by the experiments, such as the recent evolution experiments by Bell and Gonzalez [44]. The results from these experiments compare well with the analytic forecasts on evolutionary rescue [45]. For the microbial population in these experiments, the environment they live in can be manipulated by an automatic liquid processing system. Therefore, this system can be applied to verify the theory of adaptation in variable environments. For the future work, we hope that the fixation probability can be further explored both theoretically and experimentally.

## Conclusions

In this article, we expanded the classical result of the fixation probability of beneficial mutations acquired by Haldane, and calculated an approximation for the fixation probability of “generation time” mutations in a variable environment. When the selective advantage is weak, all the environmental changes are condensed into a unitary quantity: effective selective advantage. This parameter is a weighted mean across the selective advantage per generation, and the weights diminish monotonously over time. Consequently, this fixation probability in changing environments is decided by the environments that the population experienced and the average influence.

We employed our result to four attractive biological cases, which are the monotone increase of selection, the periodic change of selection, varying population size, and stochastic fluctuations in selection. In these situations, our approximation is in good accordance with the precise value, which certifies the effectiveness of our result.
